# Evaluation of Surgical Approaches to Management of Traumatic Posteromedial Elbow Instability Syndrome: A Systematic Literature Review Protocol

**DOI:** 10.7759/cureus.47880

**Published:** 2023-10-28

**Authors:** Carlos Moreno-Castillo, José Tomás Echeverría, Felipe T Martinez, Felipe Reinares

**Affiliations:** 1 Traumatology, Dr. Víctor Ríos Ruiz Healthcare Complex, Los Angeles, CHL; 2 Elbow Surgery, Complejo Asistencial Dr. Víctor Ríos Ruiz, Los Angeles, CHL; 3 Investigación Clínica, Concentra Educación e Investigación Biomédica, Viña del Mar, CHL; 4 Medicina Interna, Escuela de Medicina, Universidad Andrés Bello, Viña del Mar, CHL; 5 Traumatology, Hospital Clínico Mutual de Seguridad, Santiago, CHL

**Keywords:** trauma and orthopedic surgery, varus posteromedial rotatory instability, study protocol, elbow trauma, coronoid fracture

## Abstract

Posteromedial Instability Syndrome of the Elbow (PMIE) is a condition that arises from injuries to the stabilizing structures of the elbow joint, such as the coronoid process or ulnar lateral collateral ligament. Surgical treatment is commonly performed, but there is uncertainty regarding its results. This systematic review will synthesise the available evidence regarding the efficacy of surgical approaches to PMIE.

Published and unpublished primary studies that regard outcomes of patients treated for PMIE, irrespective of approach, will be considered for inclusion. Iterative searches will be performed in the PubMed/MEDLINE, EMBASE, and Cochrane CENTRAL repositories from their inception to May 2023. Grey literature repositories will also be searched. The Cochrane risk of bias tool will be used to assess the quality of eligible interventional studies, while the MINORS tool will be employed for observational studies. If possible, a meta-analysis based on the random-effects model will be conducted. Heterogeneity will be assessed using Cochrane's Q and I^2^ statistics, and explored through subgroup analyses and sensitivity analyses. Relevant outcomes will include elbow joint functionality as assessed by validated scales, the proportion of patients returning to usual daily life activities, the development of recurrent joint instability in the postoperative period, quality of life and the development of postoperative arthritis. This protocol has been registered in the International Prospective Register of Systematic Reviews (PROSPERO) at the University of York. Its registration number is CRD42023451516.

## Introduction and background

The elbow is a congruent joint with a high degree of stability provided by its bony structures and soft tissues [1-3]. Despite this, traumatic dislocation of the elbow is one of the most common occurrences in clinical practice, with an incidence of approximately five to six people per 100,000 adults per year. These events usually happen among young adults participating in sports, but they can also be seen in older women who experience low-energy falls. Injuries to the stabilising structures of the elbow joint can lead to the development of joint instability syndrome, for which two syndromic forms are known: posteromedial and posterolateral elbow instability (PMIE and PLIE). The latter corresponds to a relatively new entity, described in 2003 by O’Driscoll and colleagues [4], and accounts for about 10% of patients with elbow joint instability syndromes [5].

The elbow joint consists of three basic bony components [1,5], including the ulnohumeral, radiocapitellar, and proximal radioulnar joints. Within these structures, the coronoid process is particularly relevant for maintaining elbow joint stability [6,7]. This process serves as the insertion site for collateral ligaments, the anterior capsule, and the brachialis muscle, so its fracture is often associated with ligamentous injuries and posterior dislocation of the elbow. On the other hand, the ligamentous structures that play stabilising roles in the elbow joint include the medial and lateral collateral ligament complexes [5]. The lateral collateral ligament complex is composed of several structures [8,9], including the radial collateral ligament, lateral ulnar collateral ligament (LUCL), accessory ligament, annular ligament, and posterolateral ligament. The LUCL extends from the lateral epicondyle to the ulnar supinator crest and is particularly relevant in contributing to elbow joint stability, making it a key target in the treatment of PMIE, along with the repair of the coronoid process [2,10,11].

The typical mechanism of injury in PMIE is an axial load in the varus and pronation with internal rotation of the forearm [5]. Classically, this event occurs from a fall backward with the shoulder in maximal internal rotation and the elbow flexed [1,5]. The varus force induces LUCL disruption, which, in turn, allows for posteromedial subluxation of the elbow joint when exposed to internal rotation force. As the posteromedial force continues, the trochlea impacts the coronoid process, resulting in a classic concave fracture pattern on the anteromedial face of the coronoid process. If the injury persists, there may also be a rupture of the posterior ulnar collateral ligament, removing another relevant actor in maintaining the posteromedial rotational stability of the elbow [4,5,12].

The primary goal of PMIE treatment is to restore the ulnohumeral joint, prevent residual joint instability, and the development of post-traumatic osteoarthritis. Persistent joint instability and the rapid development of osteoarthritis in this condition explain why there is often great enthusiasm for surgical interventions that restore elbow stabilisation mechanisms [11]. Several approaches have been described in the literature for these interventions [5,13,14], including lateral collateral ligament complex repair and open reduction with internal fixation of the coronoid process fracture, suture and fixation of small coronoid lesions not treatable with plates and screws, and LUCL repair in association with the previous techniques. However, the evidence supporting these approaches is very limited, and recent reports also indicate favourable outcomes with medical treatment of this condition [15-17]. In order to synthesise the available evidence regarding surgical alternatives in PMIE treatment, a systematic review of published and unpublished studies will be conducted to inform clinical practice, particularly describing the most used techniques, their functional outcomes, and the most frequent postoperative complications.

Objectives

The main aim of this study is to consolidate existing evidence on the efficacy of various surgical approaches for treating PMIE in adults and to compare them with medical treatment, if possible. The focus is on evaluating their impact on multiple facets, including assessing elbow joint functionality through validated scales, gauging the return frequency to pre-injury activities, examining the incidence of recurrent joint instability post-surgery, quantifying the quality of life using established scales, and the development of postoperative arthritis clinically and radiologically. Additionally, as a secondary objective, this review seeks to discern the outcomes of patients who undergo routine repair of the LUCL as part of the surgical PMIE treatment, contrasting them with those receiving surgical treatment without LUCL repair.

## Review

Methodology

To fulfill the aforementioned objectives, a systematic literature review will be conducted, encompassing both published and unpublished studies regarding treatment alternatives for acute traumatic PMIE. This systematic review protocol follows the recommendations established by the Preferred Reporting Items for Systematic review and Meta-Analysis Protocols(PRISMA-P) [18] and it adheres to the standard methodological norms established by the Cochrane Collaboration [19]. This protocol has been registered at the International Prospective Register of Systematic Reviews (PROSPERO) and its registration number is CRD42023451516.

Search Strategy

A bibliographic search will be performed in the PubMed/MEDLINE, EMBASE, and Cochrane CENTRAL repositories for studies published from their inception until June 2023. No language restrictions will be applied for this search strategy. Additional information from unpublished studies will be obtained through a review of clinical trial protocol repositories (such as ClinicalTrials.gov), as well as preprint article databases for health sciences, such as Embase Preprints. The results will be summarised following the PRISMA flowchart [20].

**Figure 1:**
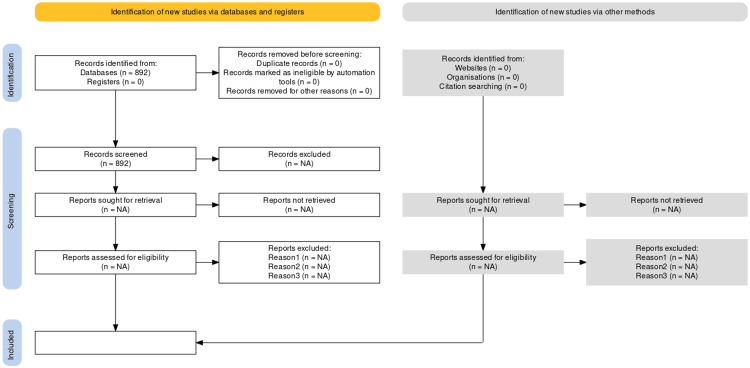
PRISMA Flow Diagram Preferred Reporting Items for Systematic review and Meta-Analysis (PRISMA) flow diagram containing preliminary results of the search strategy for this systematic review protocol on management strategies for treating patients with posteromedial rotatory instability of the elbow syndrome.

The bibliographic search will be conducted iteratively, using search terms classified under the domains Patient, Intervention, Comparator, and Outcome. The combination of these search terms will be performed using the boolean operators "AND" to combine domains and "OR" for terms within the same domain. To manage the search results, the Rayyan software [21], a web tool that facilitates duplicate detection and elimination, as well as assists in blind and independent evaluations in the process of selecting individual studies, will be used. The specific search strategy, including its terms, has been designed and conducted by a research librarian (Ms. Beata Coffey, Royal Society of Medicine) and is provided as supplementary material to this protocol in the Appendix. Preliminary results from our search strategy are also provided in Figure 1.

Only primary studies conducted on adults (>18 years old) with cases of acute PMIE treated under any therapeutic strategy, whether medical or surgical, will be considered. Acute cases of PMIE will be defined as those treated within up to four weeks after the occurrence of trauma. Given the interventional nature of the clinical question posed, studies with randomised clinical trial design or controlled clinical trials were preferentially selected to answer the research questions. However, if there are not enough primary studies that use the aforementioned designs, observational studies of prospective cohorts, retrospective cohorts, case-control studies, and case series will also be accepted for this systematic review. Letters to the editor, comments, or narrative literature reviews will not be included in the review.

The process of including primary studies will be carried out using a stepped approach. This approach involves initially selecting studies based on their relevance established through title analysis and subsequently applying a second filter to evaluate essential methodological aspects and population suitability based on abstract analysis. Potentially relevant texts will be selected from this procedure for complete evaluation. In case of discrepancies between the authors regarding the inclusion of a candidate study, the intervention of a third author (FM) will be requested as an arbiter to make the final decision on its inclusion or exclusion. The reason for the exclusion of each study will be recorded on an attached form. In addition, the reference lists of each included study will be reviewed to complement the search strategy.

Assessment of Quality of Included Studies

Once the initial screening and consideration of the aforementioned inclusion criteria are completed, all studies will be evaluated by two reviewers (CMC and JTE) in terms of their methodological quality. In case of not achieving consensus regarding an individual study's methodological quality, a third author will act as an arbiter to resolve the evaluation (FM). Given the broad spectrum of included studies, a strategy for differential evaluation of quality has been established based on the methodological design of the primary studies.

Randomised or controlled clinical trials will be evaluated using the criteria proposed by the Cochrane Collaboration for assessing the quality of intervention-focused studies [19]. These criteria include concealment of generated allocation sequence, level of masking used in each trial, number of patients lost during follow-up, and analysis performed under the intention-to-treat principle. On the other hand, observational studies will be evaluated using the Methodological Index for Non-Randomized Studies (MINORS) tool [22]. This scale includes the evaluation of eight essential elements and four additional elements relevant to comparative studies, which are scored from 0 to 2 points, where 0 means the element is absent, 1 point indicates the element exists but is inadequate for the study, and 2 points indicate the element is present and suitable for the study. In this way, scores can vary between 0 and 16 points for studies without a control group and 0 and 24 points for comparative studies, where higher scores indicate better methodological quality.

Certainty of the Evidence

Beyond the aforementioned critical analysis, the GRADE methodology will be implemented to assess the certainty of the available evidence regarding the treatment of patients with PMIE. This procedure will also be conducted by two independent authors (CMC and JTE), and in case of not reaching a consensus, a third author (FM) will be consulted as an arbiter. In summary, the GRADE methodology assigns a score that establishes the level of certainty of the evidence. GRADE has four levels to express this certainty, also known as quality of evidence: very low, low, moderate, and high. For example, evidence from high-quality studies, such as randomised controlled trials, begins with high quality, whereas evidence involving observational data begins with low quality due to inherent biases and residual confounding phenomena. The certainty of the evidence may increase or decrease for various reasons. Factors that decrease certainty include perceived risk of bias, inconsistency in effect magnitude due to heterogeneity, indirect estimates of clinical effect, imprecision in estimators, and the possibility of publication bias. Each of these elements deducts one or two points according to the uncertainty perceived by the reviewers. On the other hand, there are factors that increase the certainty of the evidence, such as the presence of a dose-response effect, a large effect magnitude, and the exclusion of possible residual confounding phenomena. These results will be synthesised in a summary table of findings, including the effects detected in an integrated manner.

Data Extraction

The data extraction process will be conducted in duplicate by two independent reviewers (CMC and JTE) to enhance the consistency of the extraction process. In instances of disagreements concerning the extracted data, these discrepancies will first be addressed through discussion between the researchers. If a consensus cannot be reached, a third reviewer (FM) will serve as an arbiter to determine the data to be included. The data to be extracted will be categorized into three domains. The first domain encompasses study characteristics, covering authors, country of publication, year of publication, and the methodological design employed (e.g., randomised clinical trial, controlled clinical trial, prospective cohort, retrospective cohort, case-control study, case report, or case series). The second domain elucidates participant characteristics, including the number of patients in the study, their age, sex, mechanism of injury (e.g., fall, sports injury, or other mechanisms), the chosen treatment approach (medical or surgical), follow-up duration, and the presence of a coronoid process fracture. Lastly, the third domain will compile surgical characteristics, such as surgical approach (e.g., medial by Hodgkin, medial by split-flexor carpi ulnaris (FCU), lateral by Kocher, lateral by Kaplan, double approach, or posterior), surgical technique, the chosen implant or fixation mechanism (screw, plate, or suture), the concurrent repair of the LUCL, and the use of a graft in the ligament repair, specifying whether the graft was autologous or from a donor. In instances of uncertainty or incomplete data within the included studies, an effort will be made to contact the corresponding author of each manuscript to collect the missing information via email.

Outcomes

The efficacy outcomes for this systematic review are elbow functionality, the presence of continuous or recurrent joint instability, and the proportion of people returning to their normal activities after treatment. Elbow functionality was considered a primary outcome of this systematic review. To establish this outcome, results from any validated scoring system for its measurement, such as the Mayo Elbow Performance Score (MEPS), the Broberg & Morrey Score, and the Abbreviated Disabilities of the Arm, Shoulder, and Hand (QuickDASH) score, were taken into account [23]. Briefly, the MEPS scale measures elbow function in four areas: pain (45 points), stability (10 points), range of motion (20 points), and daily functional tasks (25 points), with a score ranging from 0 to 100 points. These scores can be categorised into four levels, where 90-100 is excellent, 75-89 is good, 60-74 is fair, and 0-59 is considered poor [24,25]. On the other hand, the QuickDASH scale consists of an evaluation of 11 items to measure physical function and symptoms in individuals with musculoskeletal disorders in the upper limb, including traumatic elbow injuries [26].

Continuous or recurrent joint instability will be considered a second key efficacy outcome. This event was considered present if symptoms of instability were reported among patients (e.g., clicking, pain, subluxations, dislocations) and/or the presence of clinical signs of recurrent instability, such as a positive result in the valgus stress test.

The third efficacy outcome will be the quality of life reported by patients using validated scales for this purpose. Any tool designed for this purpose will be considered a valid measure for this outcome, including both generic and specific scales for assessing the elbow [27,28]. Finally, the development of elbow joint arthritis will also be deemed as a relevant postoperative outcome. Both clinical and imaging cases of the condition will be considered.

The safety outcomes will include the development of postoperative complications, specifically the occurrence of postoperative pain or stiffness, neuropathies, deep vein thrombosis, heterotopic ossifications, and postoperative infections.

Statistical analysis

Meta-analysis will be conducted whenever appropriate to synthesise the data. A random-effects method will be preferred, considering the non-pharmacological nature of the intervention, which may result in greater heterogeneity among individual studies. In case of no significant heterogeneity (see below), a fixed-effects model will be used for the synthesis.

For binary outcomes, an estimation of the relative risk or odds ratio, along with their 95% confidence intervals, will be calculated. Regarding continuous outcomes, such as the difference in joint functionality scale scores, the mean difference will be used as the summary statistic, along with a 95% confidence interval. If heterogeneity in the scales is used to assess the outcomes, the weighted mean difference will be used instead.

The heterogeneity of the results will be evaluated using Cochrane's Q and I^2^ statistics. The I^2^ statistic provides a measure of variation among the studies that cannot be attributed to chance and is expressed as a percentage. The levels of heterogeneity will be categorised as follows: <25% low, 25% to 50% moderate, and >50% high [29,30]. The fixed-effects model will be used to statistically summarise results when they are homogeneous (I^2^<25%), while the random-effects model will be preferred if there is high heterogeneity among the included studies. Additionally, heterogeneity will be explored through predefined subgroup analysis. Subgroup analyses are planned based on the surgical approach selected in each study, the surgical materials used in each study, the type of study (randomised vs. non-randomised), and the type of funding received (private industry vs. other funding). Publication bias will be assessed using a funnel plot and the Egger's test. All analyses will be performed using Review Manager (RevMan) software, version 5.2 (The Nordic Cochrane Centre, The Cochrane Collaboration, 2012, Copenhagen).

## Conclusions

Systematic reviews play a pivotal role in synthesising unbiased evidence on a particular topic by meticulously analysing a broad spectrum of studies. They employ rigorous methodologies to minimise bias, critically evaluate study quality, and identify research gaps. By assessing heterogeneity and consistency, they inform evidence-based practices, policy decisions, and optimal resource allocation.

The biomedical literature has recently recognised posteromedial instability syndrome of the elbow as a distinct entity. However, due to its recent identification and relatively infrequent occurrence, this condition remains inadequately explored, especially concerning its therapeutic aspects. Acknowledging the significance of restoring joint stability and enabling functional exercises in the recovery of PMIE patients, there is an ongoing debate within the literature regarding the most effective approach to achieving these goals. Several authors have advocated a surgical approach to the management of PMIE patients, but recent reports have also suggested favourable outcomes with more conservative alternatives in selected patients, such as those with minimal displacement or without ligamentous injuries. On the other hand, there is controversy regarding the potential benefits of routine repair of the LUCL in cases where the repair of coronoid process fractures is indicated, as the former is a structure of relatively lesser importance in maintaining joint stability. This uncertainty stems from the limited availability of high-quality evidence guiding the management of individuals affected by this condition. Bridging this knowledge gap necessitates conducting randomised clinical trials, which will offer valuable insights for clinicians challenged with providing care for these patients. Initiating a systematic literature review stands as a crucial initial step for future research in this domain. The findings of this review can significantly contribute to the development of effective treatment strategies for PMIE, potentially establishing the best surgical alternatives for this patient population. It will also provide key guidance for future research by highlighting knowledge gaps and priorities to be addressed in future trials.

## Appendices

**Appendix 1. **Search Strategy

**Table 1:** Search Strategy for EMBASE®, EMBASE Preprints® and PubMed/MEDLINE® databases Search strategy depicting search terms and combinations to search the Embase®,  Embase Preprints and  PubMed/MEDLINE® databases. This strategy was designed and conducted by Ms. Beata Coffey from the Royal Society of Medicine.

Set#	Searched for
S1	ti,ab,if,mesh,emb("postero-medial" or posteromedial or "posterior medial") and ti,ab,if,mesh,emb(elbow or rotatory or rotational) and ti,ab,if,mesh,emb(instabilit*)
S2	ti,ab,if(PMRI or VPMRI) and (elbow or elbows)
S3	MESH.EXACT("Joint Instability") and (MESH.EXACT("Elbow") OR MESH.EXACT("Elbow Fractures") OR MESH.EXACT("Elbow Injuries") OR MESH.EXACT("Elbow Joint")) and ("postero-medial" or posteromedial or "posterior medial")
S4	EMB.EXACT("joint instability") and (EMB.EXACT("elbow joint") OR EMB.EXACT("elbow") OR EMB.EXACT.EXPLODE("elbow angle") OR EMB.EXACT("elbow injury") OR EMB.EXACT("elbow fracture") OR EMB.EXACT("elbow dislocation") OR EMB.EXACT("elbow disease") OR EMB.EXACT("elbow stiffness")) and ("postero-medial" or posteromedial or "posterior medial")
S5	ti,ab,if,mesh,emb(elbow near/5 instabilit* near/5 syndrome)
S6	ti,ab,if,mesh,emb("antero-medial" or anteromedial or "anterior medial") and ti,ab,if,mesh,emb(coronoid*) and ti,ab,if,mesh,emb(fracture or fractures)
S7	ti,ab,if,mesh,emb(coronoid* near/5 (fracture or fractures))
S8	ti,ab,if(PMRI or VPMRI) and (coronoid* or ulna or ulnar)
S9	s1 or s2 or s3 or s4 or s5 or s6 or s7 or s8
S10	QU(surgery)
S11	MESH.EXACT.EXPLODE("Surgical Procedures, Operative") OR MESH.EXACT.EXPLODE("Arthroplasty") OR MESH.EXACT("Ulnar Collateral Ligament Reconstruction") OR MESH.EXACT.EXPLODE("Internal Fixators") OR MESH.EXACT("Surgical Fixation Devices") OR MESH.EXACT("Orthopedic Fixation Devices") OR MESH.EXACT.EXPLODE("Fracture Fixation") OR MESH.EXACT("Prosthesis Implantation") OR MESH.EXACT.EXPLODE("Arthroplasty, Replacement") OR MESH.EXACT("Bone-Implant Interface") OR MESH.EXACT("Elbow Prosthesis") OR MESH.EXACT("Bone-Anchored Prosthesis") OR MESH.EXACT("Prostheses and Implants") OR MESH.EXACT("Joint Prosthesis") OR MESH.EXACT("Bone Transplantation") OR MESH.EXACT.EXPLODE("Allografts") OR MESH.EXACT("Transplantation, Homologous") OR MESH.EXACT("Autografts") OR MESH.EXACT("Transplantation, Autologous")
S12	EMB.EXACT("surgery") OR EMB.EXACT("reconstructive surgery") OR EMB.EXACT("bone allograft") OR EMB.EXACT("prosthesis implantation") OR EMB.EXACT("implantation") OR EMB.EXACT("allograft") OR EMB.EXACT("autograft") OR EMB.EXACT("allotransplantation") OR EMB.EXACT("surgical technique") OR EMB.EXACT("surgical approach") OR EMB.EXACT("autotransplantation") OR EMB.EXACT("orthopedic surgery") OR EMB.EXACT("replacement arthroplasty") OR EMB.EXACT.EXPLODE("elbow arthroplasty") OR EMB.EXACT("arthroplasty") OR EMB.EXACT("joint surgery") OR EMB.EXACT("ulnar collateral ligament reconstruction") OR EMB.EXACT("ligament surgery") OR EMB.EXACT.EXPLODE("orthopedic surgical equipment") OR EMB.EXACT.EXPLODE("bone wire") OR EMB.EXACT("surgical wire") OR EMB.EXACT("surgical wire device") OR EMB.EXACT("orthopedic endoprosthesis") OR EMB.EXACT("endoprosthesis") OR EMB.EXACT.EXPLODE("bone prosthesis") OR EMB.EXACT("implant") OR EMB.EXACT("orthopedic prosthesis and orthosis") OR EMB.EXACT("orthopedic prosthesis") OR EMB.EXACT.EXPLODE("ulna prosthesis") OR EMB.EXACT("prosthesis material") OR EMB.EXACT.EXPLODE("elbow prosthesis") OR EMB.EXACT("prosthesis") OR EMB.EXACT("prostheses and orthoses") OR EMB.EXACT("prosthetic repair") OR EMB.EXACT("prosthetic alignment") OR EMB.EXACT("prosthetic fitting") OR EMB.EXACT("prosthesis fixation") OR EMB.EXACT("prosthetic replacement") OR EMB.EXACT("prosthetic procedure") OR EMB.EXACT.EXPLODE("fracture fixation") OR EMB.EXACT.EXPLODE("bone implant") OR EMB.EXACT.EXPLODE("orthopedic fixation device") OR EMB.EXACT("orthopedic implant") OR EMB.EXACT("orthopedic prostheses, orthoses and implants") OR EMB.EXACT.EXPLODE("bone graft") OR EMB.EXACT("bone transplantation") OR EMB.EXACT.EXPLODE("fracture reduction") OR EMB.EXACT.EXPLODE("open reduction (procedure)") OR EMB.EXACT.EXPLODE("closed reduction (procedure)") OR EMB.EXACT("ligament anchor") OR EMB.EXACT("bone implant interface") OR EMB.EXACT("joint prosthesis") OR EMB.EXACT("surgical patient")
S13	ti,ab,if(surgery or surgeries or surgical* or repair or repairs or repairing or repairation* or reconstruct* or reduction or reductions or fixation or fixations or fixator or fixators or screw or screws or screwing or plate or plates or plating or anchor or anchors or anchoring or nail or nails or nailing or pin or pins or pinning or crosspin* or wire or wires or replacement or replacements or arthroplast* or implant* or graft or grafts or grafting or endograft* or allograft* or autograft* or allotransplant* or autotransplant* or homograft* or endoprosthe* or prosthes* or prosthetic or docking or stabilizer or stabilizers or stabiliser or stabilisers)
S14	(s1 or s2 or s3 or s4 or s5 or s6 or s7 or s8) and (s10 or s11 or s12 or s13)

**Table 2:** Search Strategy for the Cochrane Collaboration Databases Search terms and combinations that were used to search the Cochrane Collaboration Databases. This strategy was designed and conducted by Ms. Beata Coffey from the Royal Society of Medicine.

ID	Search
#1	(("postero-medial" or posteromedial or "posterior medial") and (elbow or rotatory or rotational) and instabilit*):ti,ab,kw
#2	((PMRI or VPMRI) and (elbow or elbows or coronoid* or ulna or ulnar)):ti,ab,kw
#3	MeSH descriptor: [Joint Instability] this term only
#4	MeSH descriptor: [Elbow] this term only
#5	MeSH descriptor: [Elbow Fractures] this term only
#6	MeSH descriptor: [Elbow Injuries] this term only
#7	MeSH descriptor: [Elbow Joint] this term only
#8	("postero-medial" or posteromedial or "posterior medial"):ti,ab,kw
#9	#3 and (#4 or #5 or #6 or #7) and #8
#10	(elbow near/5 instabilit* near/5 syndrome):ti,ab,kw
#11	(("antero-medial" or anteromedial or "anterior medial") and coronoid* and (fracture or fractures)):ti,ab,kw
#12	(coronoid* near/5 (fracture or fractures)):ti,ab,kw
#13	#1 or #2 or #9 or #10 or #11 or #12
